# Effect of nasal irrigation on allergic rhinitis control in children; complementarity between CARAT and MASK outcomes

**DOI:** 10.1186/s13601-020-00313-2

**Published:** 2020-03-13

**Authors:** Dimitrios I. Mitsias, Maria V. Dimou, John Lakoumentas, Konstantinos Alevizopoulos, Bernardo Sousa-Pinto, Joao A. Fonseca, Jean Bousquet, Nikolaos G. Papadopoulos

**Affiliations:** 1 Allergy Department, 2nd Pediatric Clinic, Athens General Children’s Hospital “P&A Kyriakou”, University of Athens, Thivon and Levadias 1, Athens, Greece; 2 Gerolymatos Int.S.A., Krioneri, Greece; 3 CINTESIS, Center for Research in Health Technology and Information Systems, Faculty of Medicine, University of Porto, Porto, Portugal; 4 MEDCIDS, Department of Community Medicine, Information and Health Decision Sciences, Faculty of Medicine, University of Porto, Porto, Portugal; 5 MACVIA-France, Fondation Partenariale FMC VIA-LR, Montpellier, France; 6 Division of Infection, Immunity & Respiratory Medicine, Royal Manchester Children’s Hospital, University of Manchester, Manchester, UK

**Keywords:** Allergic rhinitis, Nasal irrigations, Sea-water solution, CARAT questionnaire, MASK/Allergy Diary app, Children and adolescents, Symptom score, Medication score, *Undaria pinnatifida*, *Spirulina platensis*

## Abstract

**Background:**

Nasal irrigations (NI) are increasingly used as an over-the-counter adjunctive treatment for allergic rhinitis (AR), but clinical studies on their effectiveness are limited.

**Methods:**

An open-label, controlled, non-randomized, real-life study was conducted to evaluate the effectiveness of NI with a new hypertonic solution as add-on treatment for AR. Children and adolescents with AR were prescribed symptomatic treatment. The active group also received an additional sea-water NI solution supplemented with algae extracts. The primary endpoint was symptom control, assessed by the control of allergic rhinitis and asthma test (CARAT) questionnaires. Moreover, the MASK/Allergy Diary was used to track symptoms and daily medication use that were combined in a novel total symptom/medication score (TSMS).

**Results:**

We assessed 76 patients. Overall, there was a significant improvement of CARAT results (median Z-score change of 1.1 in the active/NI group vs. 0.4 in the control group; *p* = 0.035). Among patients > 12 years old (n = 51), there was a significant improvement in CARAT10 results among participants receiving NI (21.0 to 25.5; *p* < 0.001), but not in the regular treatment group (21.5 to 24.0; *p* = 0.100). For children < 12 years old (n = 25), the ΝΙ group had significantly improved symptom control (CARATKids results: 5.0 to 2.0; *p* = 0.002), in contrast to the control group (4.0 to 2.5; *p* = 0.057). MASK data on allergic symptoms were comparable between groups. However, the NI group had lower TSMS, more days with < 20% symptoms and fewer days using symptomatic treatment (26.9% vs. 43.5%; *p* = 0.005).

**Conclusion:**

Addition of NI with a sea-water solution to regular treatment improved AR symptom control. CARAT questionnaires and MASK application can be useful outcome tools in real-life studies.

## Introduction

Allergic rhinitis (AR) is a very common manifestation of respiratory allergy, with a prevalence of up to 20–25% in Western societies. Children are commonly affected and, particularly if not appropriately treated, AR may lead to decreased quality of life and may be complicated with multi-morbidities such as otitis, sinusitis and asthma. In fact, AR is considered one of the main predisposing factors for the development of asthma [1, 2]. Symptomatic treatment consists of antihistamines and nasal steroids, while allergen immunotherapy is usually performed in more severe/persistent cases [3, 4]—in any case, AR usually requires chronic treatment, which raises safety concerns about protracted drug usage, leading to the quest for non-pharmacological approaches [5, 6].

Among add-on treatments, nasal irrigation (NI) with saline solutions appears to be useful [7–11], as it does not need medical prescription and is considered safe for long-term use [7]. Normal saline is primarily used, although studies have shown increased effectiveness with hypertonic solutions [8, 9]. Its mechanisms of action include direct mechanical cleansing of the mucosa from allergens or other particles, decrease of inflammatory and/or allergic mediators, and increase of mucociliary clearance. Meta-analyses suggest that NI can be used as add-on to medical treatment [11]. However, in children, NI is not yet part of the guideline recommendations, probably due to insufficient evidence of its pediatric effectiveness.

Therefore, this study aimed to evaluate the effectiveness of NI as an add-on treatment for AR, using a new NI product based on sea-water supplemented with (1) extracts from the sea weeds *Undaria pinnatifida* and *Spirulina platensis,* and (2) dexpanthenol. This is the first study using these compounds intranasally: *Undaria pinnatifida* is a brown alga rich in fucoidans (i.e. sulfated polysaccharides) with anti-inflammatory and anti-allergic properties [12, 13]; *Spirulina platensis* is a green alga whose active components are phycocyanins, which have multiple possible actions, including an anti-allergic effect in the respiratory epithelium [14, 15]; dexpanthenol, the alcoholic analogue of pantothenic acid, acts as a mucosal moisturizer and hydrating agent [16]. To evaluate NI effectiveness (i.e. disease control, symptoms and use of medication), we used the Control of Allergic Rhinitis and Asthma Test (CARAT) [17, 18] and the MASK/Allergy Diary app, developed by the ARIA group [19].

## Methods

### Design of the study

This was an open-label, controlled, real-life, non-randomized, prospective quasi-experimental study. The attending physician prescribed the treatment according to guidelines and the patient/caregiver chose to use (or not) supplementary NI in addition to the prescribed treatment. Therefore, an active/NI group (standard AR therapy plus NI) was compared with a control group (standard AR therapy only) regarding AR symptoms and medication use.

### Setting and patients

This study was performed at the Allergy Unit of the 2nd Pediatric Clinic, University of Athens, a tertiary referral center. We included all eligible and consenting children and adolescents (6–19 years old) with symptoms suggestive of AR (such as, but not exclusively, runny and/or blocked nose, sneezing, nasal itch) for at least 6 months, observed between April 2017 and August 2018. In all, skin prick tests and/or specific IgE confirmed sensitization to at least one relevant aeroallergen (using a standard panel consisting of seasonal and perennial allergens, including, but not exclusively, grasses, olive, birch, mugwort, ragweed, *Parietaria* and cypress pollen, *Dermatophagoides pteronyssinus* and *farinae*, *Alternaria*, cat and dog epithelia, and cockroach). Exclusion criteria consisted of adenoidal hyperplasia, septal deformity, polyps, infectious rhinitis, vasomotor rhinitis, rhinitis medicamentosa, i.e. signs and symptoms indicative of non-allergic rhinitis (e.g. not responsive to regular treatment, fever, apnoea, decongestant overuse). We also excluded children at need of oral corticosteroids, at the time of presentation.

### Performance of the study

All children were prescribed indicated rhinitis treatment, consisting of a second-generation antihistamine and/or nasal corticosteroid (CS) or CS + azelastine. All patients/parents were instructed in allergen avoidance measures and in the nasal spray (and irrigation, if applicable) technique and were instructed to use pharmacological treatment for 10-15 consecutive days, resuming it in case of persisting symptoms. Mast cell stabilizing eye drops were prescribed for conjunctivitis refractory to antihistamines, and asthma was treated with inhaled CS or CS + long-acting β-agonist, according to the guidelines. A follow-up visit was planned for 30 days later. Signed informed consent was obtained by all patients/parents. This study was approved by the Ethics Committee of the “P.& A. Kyriakou” Children’s Hospital.

### Intervention: nasal irrigations

The active/NI group received a nasal spray consisting of sea-water hypertonic (2.3% NaCl) solution with extracts from *Undaria pinnatifida* and *Spirulina platensis* algae and dexpanthenol (“Sinomarin + Algae Allergy Relief”, available as over-the-counter). The patient/caregiver was instructed to use the product daily during the observation period according to the manufacturer’s instructions: 2 puffs/nostril 3 times/day for children < 12 years old; 4 puffs/nostril up to 5 times/day for children > 12 years old. Nasal irrigations were performed at least 15 min before potential inhaled CS use. However, adherence either to pharmacologic treatment or NI was not further enforced or assessed. Nevertheless, prospective data on medication use were gathered and analyzed through the MASK/Allergy Diary as described in detail below.

### Outcomes

#### CARAT questionnaires

Control of allergic rhinitis and asthma test (CARAT) is a brief self-administered questionnaire aiming to quantify symptoms and control of AR and asthma. There are two versions of CARAT—(i) CARAT10 was used by participants > 12 years old, who filled it directly (as it has been designed for adults and teenagers), and (ii) CARATKids, which was used by participants aged 6–12 years old and filled by them together with their parents.

CARAT10 assesses the previous 4 weeks. The responses for all ten questions are on 4-point Likert scales, scoring from 0 to 3. The final score ranges from 0 to 30, with scores > 24 indicating good control of asthma and AR [17], and four-point changes being considered clinically relevant [20]. CARAT10 has been translated and validated to Greek (kindly provided by Prof. I. Tsiligianni).

CARATKids includes 13 “Yes/No” questions: affirmative answers scored as 1 (corresponding to “symptom/item present”), while negative answers scored as 0 (“symptom/item not present”). Therefore, 13 points correspond to a complete absence of control [18]. A version translated by the authors was used.

#### MASK/Allergy Diary application

Allergy diary is a free, on-line application for smartphones, developed by MASK-rhinitis (Mobile Airways Sentinel NetworK for AR) to evaluate AR symptoms and disease control [19, 21, 22]. It contains four visual analogue scales (VAS) measuring nasal, ocular, asthma and overall allergic symptoms. It is used prospectively and filled daily, allowing also for registering the medications used each day [23]. The application does not integrate symptom and medication scores [24] and, therefore, we calculated a Total Symptom/Medication Score (TSMS) based on equal weight, as suggested by EAACI [25]: ${\text{TSMS}}\, = \,\frac{{{\text{Symptom}}\;{\text{Score}} + {\text{Medication}} \;{\text{Score}}}}{2}$, where symptom score is the VAS 0-100 score, and Medication Score is a 0-100 score with the following punctuation system depending on the AR medication taken: no medication = 0 points; oral and/or topical (eyes or nose) H1antihistamines $\left( {\text{H1A}} \right)\, = \,100 \times \frac{1}{3}$ points; intranasal CS (INS) with/without ${\text{H1A}}\, = \,100 \times \frac{2}{3}$ points; oral CS (with/without INS and with/without H1A) = 100 points. Of note, this approach is based on the clinical effects of pharmacotherapy on symptom reduction, but neither takes into account the use of specific medication (only the drug class) or daily dosing nor has yet been validated [25]. Therefore, apart from the 4 Symptoms Scores (nasal, ocular, asthma and overall allergic symptoms VAS), 4 TSMS (i.e. taking into account daily medication) were established. “Symptom-free days” were defined as those with score < 20% (either for VAS/Symptom Score or TSMS) [23]. All patients/parents were trained to use MASK application and upload data accordingly.

### Statistical analysis

Continuous variables were described using medians and percentiles 25–75, while categorical variables were described with absolute frequencies and percentages. CARAT and MASK/Allergy Diary results were compared using Wilcoxon’s rank-sum and Kruskal–Wallis tests (continuous variables), and Pearson’s Chi squared test (categorical variables). Intention to treat analyses were both performed altogether for participants of all ages, and separately for children 6–12 and > 12 years old.

To perform a combined analysis on CARAT10 and CARATKids results, Z-scores were separately computed for CARAT10/CARATKids and, for each, both reference values and standard-deviations of all values (i.e. those collected at first visit and at day 30) were considered. Z-scores changes (i.e. Z-scores differences between days 1 and 30) were compared between the active and control group using Mann–Whitney U test. Additionally, to test the consistency of our results, a non-parametric ANCOVA was performed, with day 30 Z-scores as dependent variable and day 1 Z-scores as co-variate. To assess whether CARAT10 and CARATKids results are sufficiently similar to be assessed together, a meta-analysis of standardized mean changes was performed—an *I*^2^ > 50% and a Cochran Q-test *p*-value < 0.10 were considered to represent substantial heterogeneity. Statistical analysis was accomplished with R software, version 3.5.0. Significance level was defined at 0.05.

The RELEVANT criteria were considered in the design and reporting of this study [26]. RELEVANT is a recently developed tool by Respiratory Effectiveness Group (REG) and EAACI, designed to evaluate the quality of real-life studies and whether they may be used in guidelines.

## Results

**Fig. 1:**
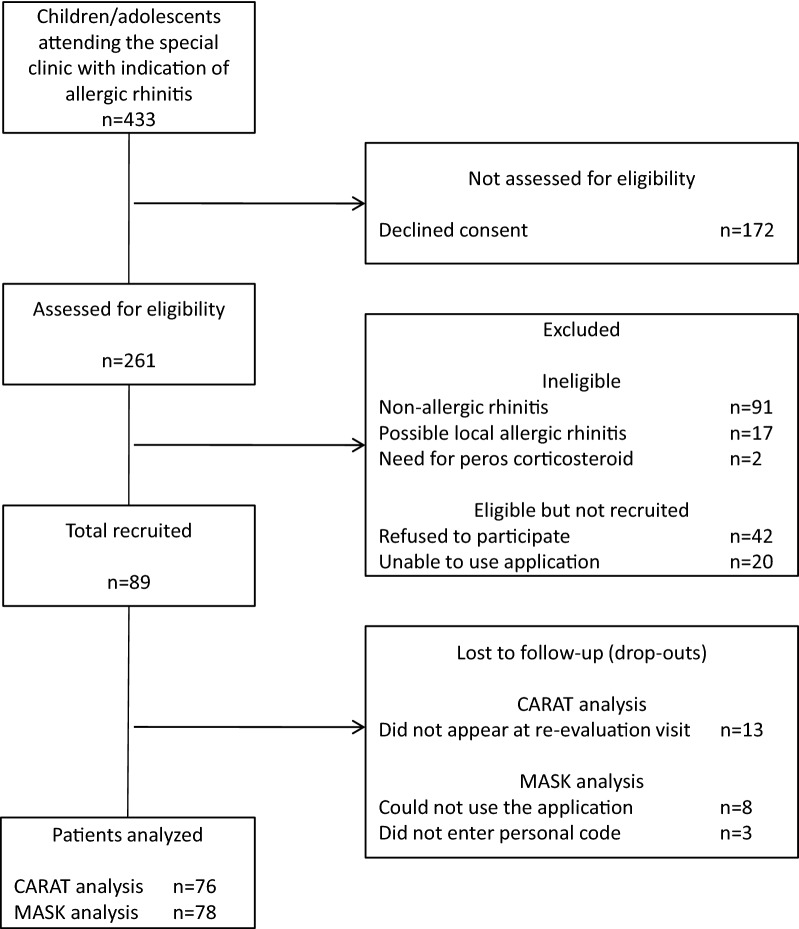
Flow chart of the selection process of included participants

Overall, 89 children and adolescents with symptoms suggestive of AR agreed to participate in this study. Seventy-six children (85%) returned for the second visit (for whom we present CARAT data) (Fig. 1). Of those, 53 children used NI (active group), while 23 did not use NI (control group). Children in the active/NI group, controls and drop-outs did not differ demographically, clinically or in initial CARAT scores (Tables 1, 2). The MASK Allergy Diary application was used by 78 children (88% acceptance rate, average of 19.7 entries/child), corresponding to 55 children in the active group and 23 controls.

**Table 1:** Characteristics of the recruited study participants, as well as of the participants with complete CARAT questionnaire and MASK/Allergy Diary results

	Recruited participants (active/control group)	*p*-value *	CARAT outcome group (active/control group)	*p*-value *	Allergy diary outcome group (active/control group)	*p*-value *
Total	89 (64/25)		76 (53/23)		78 (55/23)	
Age		0.592		1.000		0.520
> 12 years old	59(44/15)		51 (36/15)		50 (37/13)	
< 12 years old	30(20/10)		25 (17/8)		28 (18/10)	
Gender		0.578		0.427		0.485
Male	62 (43/19)		53 (35/18)		55 (37/18)	
Female	27 (21/6)		23 (18/5)		23 (18/5)	
Asthma^a^	31 (24/7)	0.550	26 (19/7)	0.846	28 (22/6)	0.363
Sensitizations		0.929		0.873		0.819
1	20 (14/6)		14 (9/5)		17 (11/6)	
2	31 (22/9)		27 (19/8)		28 (20/8)	
> 2	38 (28/10)		35 (25/10)		33 (24/9)	

* Pearson’s Chi square test

^a^Concurrent allergic asthma

**Table 2:** CARATKids and CARAT10 scores on day 1, and individual differences between day 1 and day 30: CARAT scores in day 1 did not differ significantly for active, control and drop-outs; by contrast, CARAT10 changes for each patient were significantly different between day 1 and day 30

	Active (n: 53)	Control (n: 23)	Drop-outs (n: 13)	*p*-value
CARATKids (score range 0–13)	n: 17	n: 8	n: 5	
Day 1	5.0 (3.8–6.3)	4.0 (3.0–7.0)	5.0 (4.0–5.3)	0.878 *
Day 1/day 30 difference	− 3.0 (− 5.0 to 2.0)	− 2.00 (− 4.0 to 0)		0.574 **
CARAT10 (score range: 0–30)	n: 36	n: 15	n: 8	
Day 1	21.0 (17.0–23.0)	21.5 (17.5–24.0)	19.0 (17.0–22.0)	0.536 *
Day 1/day 30 difference	5.0 (0.8–6.3)	1.00 (0.5–2.5)		0.023 **

*p*-values < 0.05 are shown in italics

Values shown as medians with 25–75 percentiles

* Kruskal–Wallis test

** Wilcoxon’s rank-sum test

### Disease control

#### CARAT10

**Fig. 2:**
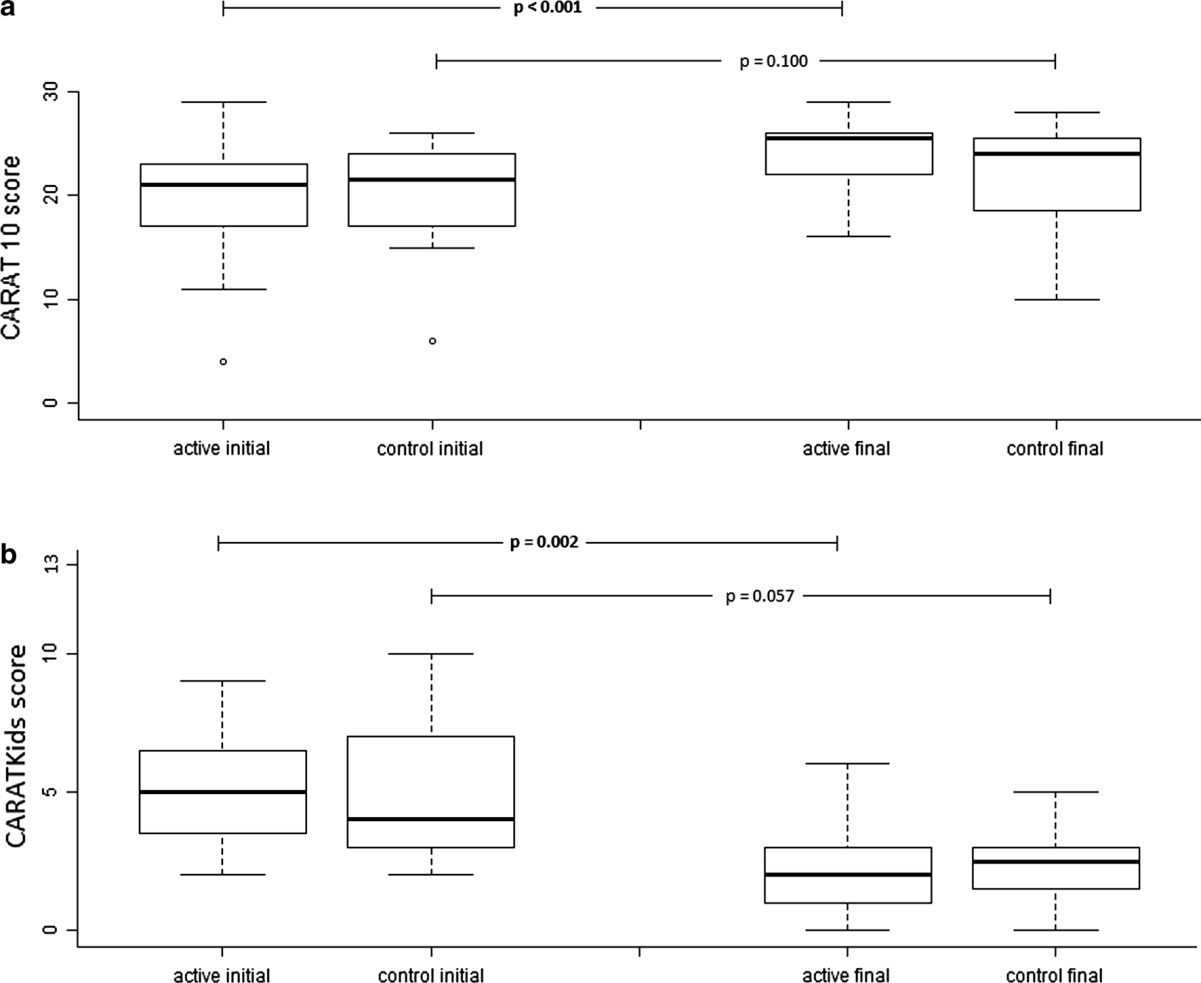
Initial (day 1) and final (day30) CARAT scores for (**a**) adolescents > 12 years old (CARAT10), and (**b**) children < 12 years old (CARATKids). Analysis was performed with Wilcoxon’s signed-rank test

We assessed 51 participants aged > 12 years old, with those on the NI group ending up with significantly improved disease control compared to those on the control group. In fact, when we compared day 1 to day 30 scores, for the active group, day30 median CARAT10 score was 25.5 (25–75 percentiles: 22.0–26.0), up from 21.0 (25–75 percentiles: 17.0–23.0) on day 1 (*p* < 0.001); on the other hand, for the control group, median day 30 score was 24.0 (25–75 percentiles: 18.5–25.5), up from 21.5 (25–75 percentiles: 17.5–24.0) on day 1 (*p* = 0.100) (Fig. 2a). Moreover, the improvement of CARAT10 score (i.e. the individual differences between day 1 and day 30) was significantly higher for the active than for the control group (median increase: 5.0 vs. 1.0, respectively; *p* = 0.023) (Table 2). This difference surpassed the limit of 3.5 that is considered by the CARAT developers (also co-authors of the present study) to be the minimal clinically relevant difference [20] and was achieved despite the fact that both groups had (i) similar instructions in regard to regular pharmaceutical treatment, and (ii) similar severity of symptoms on day 1.

#### CARATKids

Twenty-five children < 12 years old completed the CARATKids questionnaire. Day 30 median CARATKids score for the active group was 2.0 (25–75 percentiles: 1.0–3.0), down from 5.0 (25–75 percentiles: 3.8–6.3) on day 1 (*p* = 0.002); for the control group, the day 30 score was 2.5 (25–75 percentiles: 1.8–3.0), down from 4.0 (25–75 percentiles: 3.0–7.0) on day 1 (*p* = 0.057) (Fig. 2b). However, the 30-day differences in CARATKids values between active and control groups were not significantly different (median decrease of − 3.0 vs. − 2.0; *p* = 0.574) (Table 2).

#### Combined analysis of CARAT10 and CARATKids

**Fig. 3:**
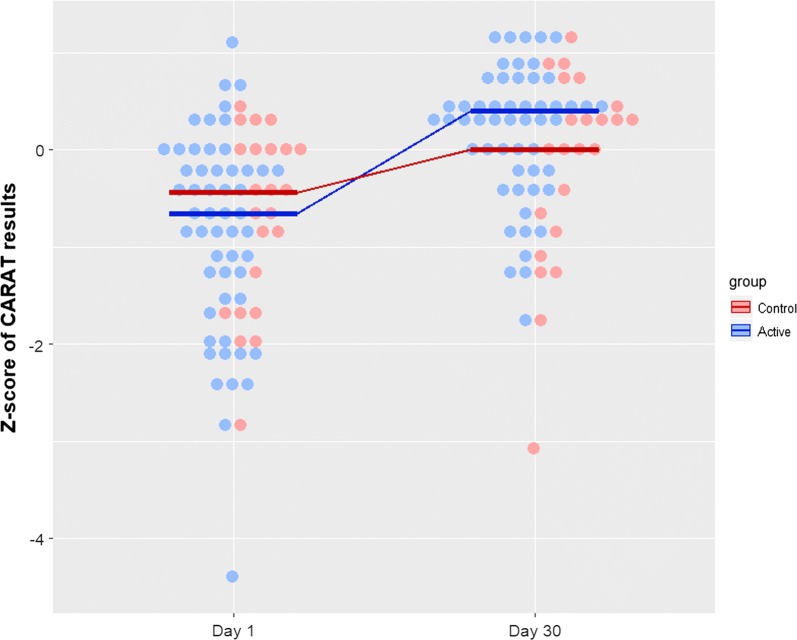
Dot plot for the Z-scores of CARAT10/CARATKids results for active and control groups on day 1 and day 30

**Fig. 4:**
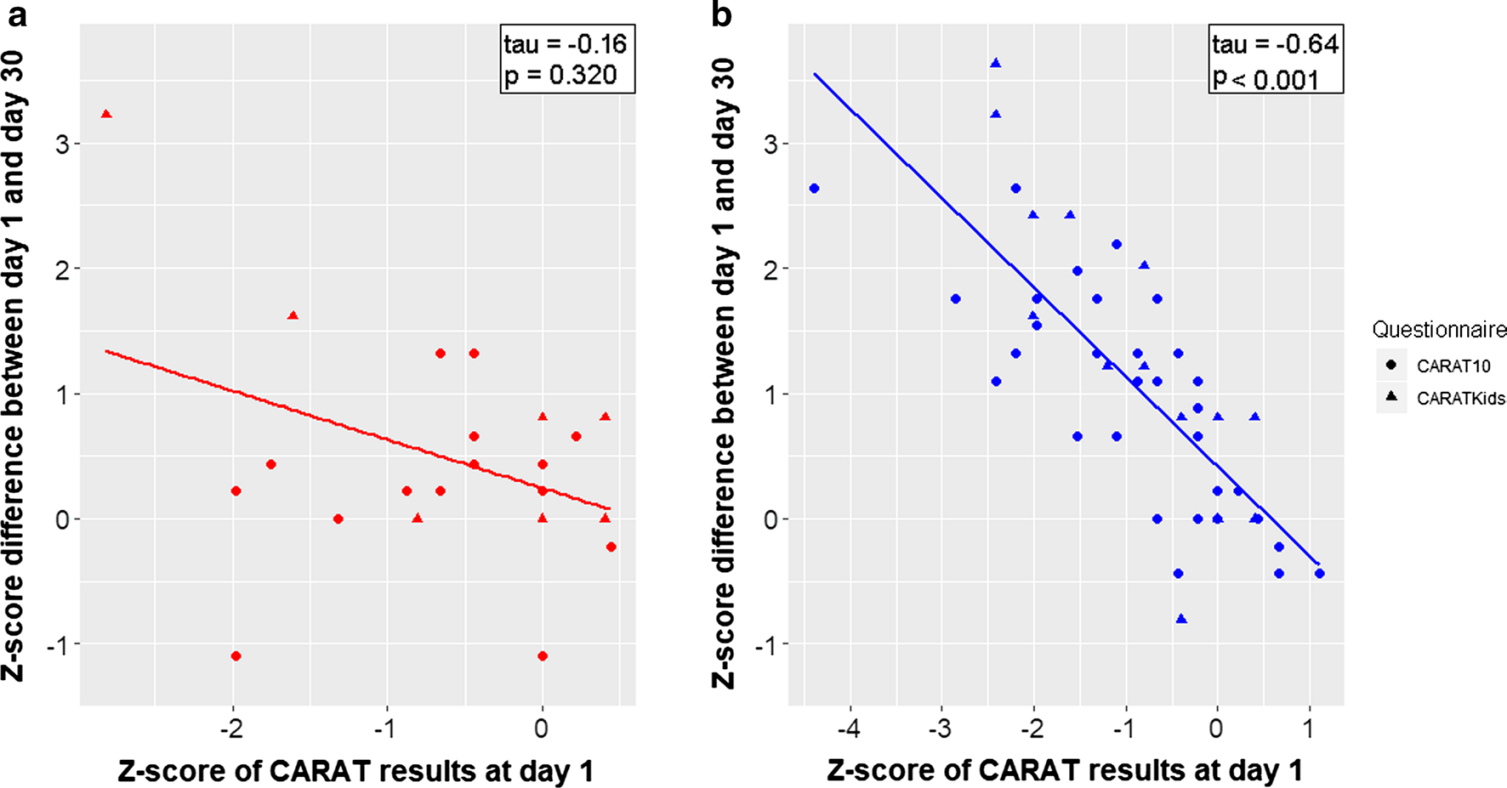
Correlation of initial (day1) CARAT Z-scores and 30-days Z-score difference in the control group (**a**) and in the active group receiving nasal irrigations (**b**)

To analyze the results of all participants together, we computed Z-scores for CARAT10 and CARATKids. The active group presented a significantly higher improvement on its Z-score compared to the control group (median improvement: 1.1 vs. 0.4 points, respectively; *p* = 0.035) (Fig. 3). These results were consistent to those obtained when performing a non-parametric ANCOVA comparing day 30 Z-scores between the active and the control group, adjusted for day 1 Z-scores (*p* = 0.004). Of interest, the lower the initial Z-score (i.e. the lower the initial symptom control), the higher was the difference at the end (i.e. the greater the benefit for the patient; Fig. 4).

**Fig. 5:**
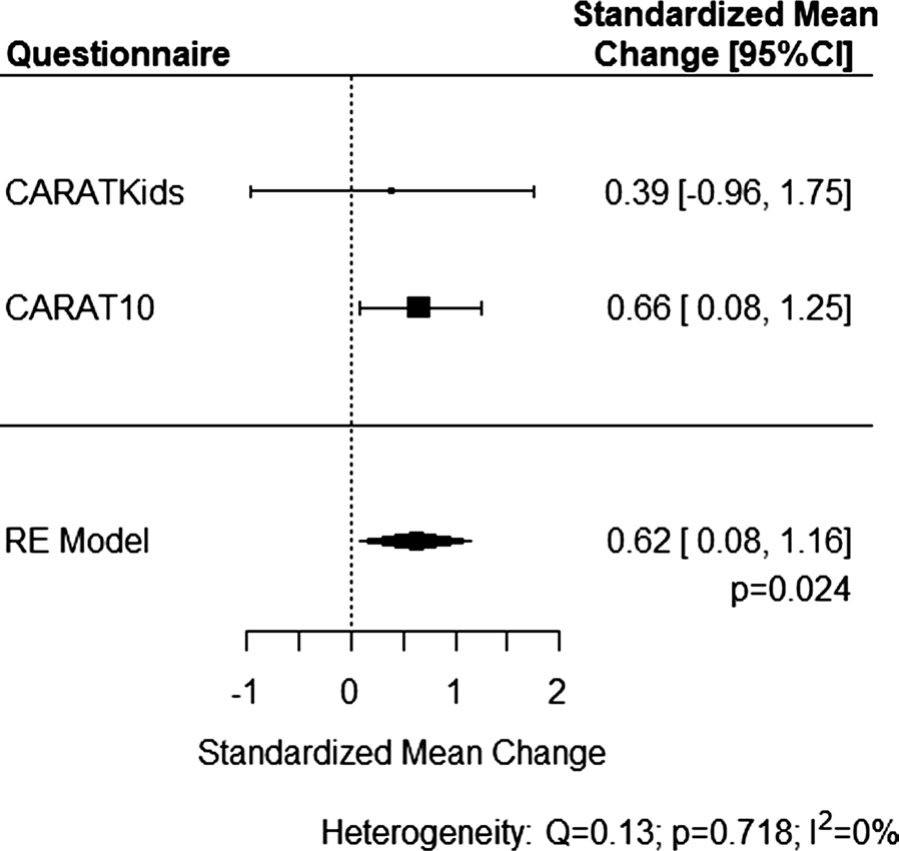
Meta-analysis of standardized mean changes (between day 1 and day 30) of CARAT results, pooling CARATKids and CARAT10 results

Finally, we performed a meta-analysis pooling CARATKids and CARAT10 results. The standardized mean change in CARAT results was significantly higher in the active than in the control group (0.62, 95% CI 0.08–1.16; *p* = 0.024), with no heterogeneity observed (*I*^2^ = 0%; *p* = 0.718) (Fig. 5).

### Symptoms and medication

There were no significant differences in symptom scores as evaluated by the VAS. However, when TSMS was calculated, significant differences emerged for all 4 parameters (Table 3). When medication use was taken into account (i.e. TSMS), children using NI were symptom-free (days with < 20% on VAS) for significantly more days regarding all parameters except the ocular one (Table 4).

**Table 3:** Ocular, nasal, asthma and overall allergic symptoms using Visual Analogue Scales (VAS) for symptoms and total symptom and medication score (TSMS) (i.e., considering medication use on the respective days)

	Active (n: 55)	Control (n: 23)	*p*-value*
Ocular symptoms^a^	1.3 (0.1–8.5)	4.1 (0.2–10.3)	0.482
Nasal symptoms^a^	20.0 (9.2–27.6)	20.6 (9.0–28.7)	0.936
Asthma symptoms^a^	0.4 (0.0–9.5)	0.1 (0.0–3.0)	0.503
Overall allergic symptoms^a^	18.0 (8.7–26.3)	18.6 (11.6–27.8)	0.701
Ocular TSMS^b^	9.5 (6.5–17.4)	15.7 (12.2–23.6)	*0.019*
Nasal TSMS^b^	18.8 (12.9–22.3)	23.6 (17.3–28.0)	*0.046*
Asthma TSMS^b^	10.8 (6.6–16.8)	15.6 (11.1–20.4)	*0.039*
Overall allergic TSMS^b^	17.6 (13.0–22.1)	23.6 (14.1–28.2)	*0.035*

*p*-values < 0.05 are shown in italics

Values shown as medians and 25–75 percentiles

* Wilcoxon’s rank-sum test

^a^Mean daily VAS

^b^Mean daily TSMS

**Table 4:** Percentage of symptom-free days, regarding different types of symptoms

Percentage (%) of symptom-free days	Active (n: 55)	Control (n: 23)	*p*-value *
Ocular symptoms^a^	100.0 (85.2–100.0)	96.8 (81.0–100.0)	0.483
Nasal symptoms^a^	67.7 (50.0–87.9)	68.0 (44.8–85.7)	0.896
Asthma symptoms^a^	100.0 (87.5–100.0)	100.0 (96.6–100.0)	0.540
Overall allergic symptoms^a^	69.6 (40.9–90.7)	75.0 (41.7–85.7)	0.906
Ocular TSMS^b^	75.0 (53.7–85.7)	66.7 (46.2–77.3)	0.054
Nasal TSMS^b^	64.5 (50.8–76.8)	44.0 (38.5–56.5)	*0.019*
Asthma TSMS^b^	76.9 (61.8–85.0)	64.0 (42.9–76.2)	*0.016*
Overall allergic TSMS^b^	65.4 (51.9–77.8)	42.9 (36.7–61.5)	*0.012*

*p*-values < 0.05 are shown in italics

Values shown as medians with 25–75 percentiles

Corresponding to < 20% on ^a^ visual analogue scales (VAS) for symptoms and ^b^ Total Symptom and Medication Score (TSMS)

* Wilcoxon’srank-sum test

Interestingly, the improvement in the active group was achieved with less medication use: (i) children using ΝΙ received the prescribed pharmacologic treatment for significantly less days, when compared to the control group (median: 26.9% vs. 43.5% of the days, *p* = 0.005) and (ii) the active group used less medication as judged by the calculated Medication Score (median: 16.7 vs. 25.7, *p* = 0.006) (Table 5).

**Table 5:** Medication use in subjects using nasal irrigation (active group) and in the control group, as assessed by the percentage of days on treatment and by the medication score

	Active (n: 55)	Control (n: 23)	*p*-value *
Percentage (%) of days on treatment			*0.005*
Median (25–75 percentile)	26.9 (19.1–40.7)	43.5 (31.0–61.2)	
Medication score^a^			*0.006*
Median (25–75 percentile)	16.7 (10.7–25.0)	25.7 (17.3–35.7)	

*p*-values < 0.05 are shown in italics

^a^Medication score 0**–**100 depending on the drugs taken

* Wilcoxon’s rank-sum test

## Discussion

In this proof-of-concept study, a hypertonic sea-water (2.3% NaCl) based solution supplemented with extracts from the sea weeds *Undaria pinnatifida* and *Spirulina platensis* and dexpanthenol was found to be effective as an add-on treatment in symptom control of children with AR, suggesting that NI may improve AR control with reduced use of medication.

The medications commonly used for AR have shown by objective measures to be effective and safe even for prolonged use. However, regarding children, parents/caregivers are often skeptical and unwilling to follow long-term treatments, which results in considerably low adherence [27]. Nasal irrigations (particularly those with normal saline) have long been used to remove mucus from the upper respiratory tract, as they are safe, tolerable and more easily acceptable by parents/caregivers. The effectiveness of NI not only includes mechanical removal of mucinous excretions, but also an increase of mucociliary function, decreased interaction of allergens with the nasal mucosa [8], and reduced release of inflammatory mediators such as histamine, prostaglandins and leukotrienes and/or receptors (such as ICAM-1) [28]. Studies have shown their efficacy in AR and sinusitis, and meta-analyses support their use [11, 29], with hypertonic solutions (e.g. 2.3% NaCl) being apparently more effective than normal saline solutions [8, 9, 30].

This is the first study assessing the intranasal use of algae, with the species used being known for having, among other, anti-allergic effects [12–15]. It is also the first study in children and adolescents with parallel, two way evaluation of the effectiveness of NI; intention to treat analysis was performed.

We used a combination approach to evaluate the effectiveness of NI, namely involving the use of CARAT questionnaires and MASK/Allergy Diary application. Concurrent use of data from many sources is encouraged so as to provide a wider angle of the impact of AR in each patient [31]. CARAT gives a retrospective view on the symptoms occurred over the last weeks, while the MASK/Allergy Diary provides prospective daily data on symptoms and on medication use, giving the opportunity to longitudinally follow the course of AR. Although this study did not aim to formally compare CARAT and MASK/Allergy Diary outcomes, our results indicate that these tools can be used in parallel, augmenting the ability to clinically assess AR activity.

According to our results, NI can be helpful as an add-on treatment in children with allergic rhinitis. The effect seems to be more prominent in adolescents, possibly reflecting several factors, including different pathophysiology, different volume of NI, difficulty in symptoms monitoring in younger patients, and—probably most important—the smaller sample size regarding patients < 12 years old.

When specific allergic symptoms were assessed there was some discordance between CARAT (showing considerable decrease between start and end of the evaluation) and the Allergy Diary (that showed no difference in symptom score alone, according to VAS, in the longitudinal follow-up). This may reflect a rapid improvement of symptoms after the initial evaluation, leading to a dilution of the effect over a 30-day period. However, when medication use was integrated with VAS (i.e. TSMS), it became apparent that control patients regulate their symptoms by treatment use [32] as they used more medication (antihistamines and/or nasal steroids); this was not the case of the active group, whose patients managed to have less symptoms with less pharmacologic treatments. Therefore, our newly suggested MASK/Allergy Diary-based TSMS appears to be a good descriptor of respiratory allergy control. Of note, as with all Medication Scores used up to date, there is no differentiation between specific compounds of the same class (i.e. different H1A and/or intranasal CS). Accordingly, to track efficacy differences among drugs of the same class was out of the scope of our study.

Our results may suggest that TSMS complements CARAT, as we observed lower use of pharmaceutical treatment concurrent with an improvement of disease control in the NI group. This is one of the first studies using the Allergy Diary for a longitudinal cohort study, in what appears to be a valuable tool, with unexplored potential.

### Limitations

Our study has some limitations, as it was non-randomized and assessed a relatively small sample (particularly for the lower age group). In fact, the low number of children < 12 years old might explain why some differences in that group were not found to be statistically significant. In addition, blinding in our study was not feasible and the placebo effect of NI cannot be excluded. However, while the evidence level is not that of a randomized trial, our conclusions offer insight into the use of medication and real-life effectiveness of different treatment modalities—our participants adhered variably to prescribed pharmacological treatment but adhered well to more “natural” NI and still achieved improved symptom control. Such an insight would have been impossible in the context of a randomized control trial. Another limitation is that the TSMS presented herein, based on MASK/Allergy Diary [23] and on the EAACI Position Paper [25], had not been formally validated. However, both VAS and a Combined Symptom/Medication Score were recently validated [33]. Therefore, given the heterogeneity of the several scores that attempt to merge symptoms and medication, the use of a simple, commonly used Medication Score as the one suggested in this study should be encouraged. It appears, therefore, that the use of a novel TSMS concept deserves further consideration.

Another limitation stems from the impossibility of knowing whether the observed beneficial effect of NI can be attributed to the hypertonic sea-water, the “active” compounds, or both; in fact, the increasing number of products with different compositions available prompts the need for studies separating class effect from individual product effects. However, assessing that would require a much larger sample and a different study design, which would be practically impossible to perform taking into account the logistical considerations of a real-life study. Additionally, further studies are required to assess adults and patients with other rhinitis phenotypes, to whom our results cannot be generalized. By contrast, as this study was performed during a 14-month period and included both seasonal and perennial AR, it is possible that the presented results maybe generalized to different AR triggers.

In summary, ΝΙ with sea-water, supplemented with sea weed extracts and dexpanthenol, was found to be effective as an add-on therapy for children and adolescents with AR. Improved symptom control was achieved with reduced medication use in a real-life setting. Additional studies are needed to evaluate the potential role of NI in the algorithm for AR treatment. In this context, tools such as the CARAT questionnaires and the MASK/Allergy Diary, particularly when used together, can collect important data and guide future guidelines.

## Data Availability

The datasets used and/or analyzed during the current study are available from the corresponding author on reasonable request.

## Abbreviations

AR: Allergic rhinitis
NI: Nasal irrigations
CARAT: Control of Allergic Rhinitis and Asthma Test
MASK: Mobile Airways Sentinel Network
TSMS: Total Symptom and Medication Score
RELEVANT: REal Life EVidence AssessmeNt Tool
